# 

**Published:** 1893-02

**Authors:** 


### CONTENTS.

ORIGINAL ARTICLES—General Treatment of Fractures, a Clinical Lecture by F. S. Dennis, M. D., New York City .......... 56
Dislocations of the Shoulder; By William Meacher, M. D., Portage, Wis. 60
Spina Bifida Occulta; By A. Bowen, M. D., Nebraska City, Neb....... 68
CORRESPONDENCE—New York Letter.......... 71
SOCIETY REPORTS—The Clinical Society of Maryland.......... 75
Gynecological and Obstetrical Society of Baltimore.......... 83
BOOK REVIEWS—Manual of Practical Medical and Physiological Chemistry, by Chas. E. Pellew, E. M.......... 88
Genito-Urinary and Venereal Diseases, by Chas. H. Chetwood, M. D... 89
Materia Medica and Therapeutics, by L. F. Warner, M. D.......... 89
Diseases of Children, by C. Alexander Rhodes, M. D.......... 89
Practice of Medicine, by Edwin T. Doubleday, M. D.......... 89
SELECTIONS AND ABSTRACTS—Successful Cases best Treated by Successful Remedies.......... 90
Expert Testimony.......... 92
Affections of the Testicle in Hereditary Syphilis.......... 93
A New Method for Artificial Respiration in Asphyxia Neonatorum...... 94
On the Radical Treatment of Certain Cases of Grave Concealed Accidental Hemorrhage.......... 95
Double Penis—Absent Anus.......... 95
Are Hypodermic Injections of Morphine Lawful?.......... 96
A Rare Aural Complication of Gonorrhea.......... 97
To Remove Nitrate of Silver Stains from the Fingers.......... 97
EDITORIAL.—Serious Consequences of Perforation of Appendix.......... 98
EDITORIAL NOTES.......... 100
BOOKS AND PAMPHLETS RECEIVED.......... 103
PRESCRIPTION DEPARTMENT.......... 104
SPECIAL NOTES.......... 106

